# A scoping review of applications of natural language processing for chronic pain research

**DOI:** 10.1007/s44163-026-01417-7

**Published:** 2026-07-02

**Authors:** Swati Rajwal, Selen Bozkurt, Jeanmarie Perrone, Anne Marie McKenzie-Brown, Abeed Sarker

**Affiliations:** 1 Department of Biomedical Informatics, Emory University School of Medicine, Atlanta, GA 30322 USA; 2 Department of Emergency Medicine, Perelman School of Medicine, Philadelphia, PA 19104 USA; 3 Department of Anesthesiology, Emory University School of Medicine, Atlanta, GA 30307 USA

**Keywords:** Chronic pain, Natural language processing, Language models, Text mining, Scoping review

## Abstract

**Background:**

Chronic pain poses a significant global health burden, with pertinent contextual information relevant to it encapsulated in free text format across sources such as electronic health records (EHRs), published literature, and social media. Natural language processing (NLP), including recent advances in large language models (LLMs), presents a transformative opportunity to analyze this unstructured data, but the literature is fragmented across disciplines, and there is a need to consolidate existing knowledge, identify gaps in the literature, and inform future research directions in this emerging field.

**Objective:**

This review aims to investigate and characterize NLP-based methods designed for chronic pain research.

**Methods:**

A structured search was conducted across five databases (PubMed, Web of Science, Scopus, IEEE Xplore, ACL Anthology) for English-language studies published between 2014 and 2025. Included studies were analyzed for design, research question, data sources, NLP methods, population characteristics, and reproducibility.

**Results:**

From 155 records, 34 met inclusion criteria. The majority of studies (23/34; 67%) were published in the past three years. Most studies (12/34) relied on EHR data, with others utilizing social media data (n=7), synthesizing literature (n=2), or leveraging other data sources. Earlier studies focused on rule-based methods, while recent work adopted transformer-based models (e.g., BERT, RoBERTa, BioBERT) and LLMs (e.g., GPT-3.5 for zero- or few-shot learning frameworks). Common challenges included limited dataset diversity, lack of standardized evaluation frameworks, and poor reproducibility practices.

**Conclusions:**

While the application of NLP for chronic pain research is promising, the review revealed a paucity of research on the topic, with opportunities for future explorations such as validation of generalizable approaches involving diverse data and cohorts, multimodal data validation systems, public release of data and models, and the development of standardized evaluation metrics to enhance reproducibility and equity in chronic pain research. The emerging use of NLP offers clinicians new ways to observe patient needs, communicate more clearly, and make pain care more responsive and humane.

**Supplementary Information:**

The online version contains supplementary material available at https://doi.org/10.1007/s44163-026-01417-7.

## Introduction

Chronic pain, defined as pain that persists or recurs for three months or more [1], is a major health problem in the United States (US), with over 30% of the adult population estimated to suffer from it in some form [2–4]. It poses a substantial financial burden as well, with associated annual costs estimated to be between $560 and $635 billion [5–8]. These estimates place the financial burden higher than that for diabetes, cancer, and cardiovascular diseases [3, 6–8]. The burden of chronic pain is compounded by its frequent co-occurrence with significant anxiety or depression; over half of those with chronic pain also report persistent mental health symptoms, and nearly 70% say health issues limit their work, daily tasks, and social activities [9]. Chronic pain presents a similar health problem globally, particularly in low- and middle-income countries, with a prevalence estimated at 33% among all adults and 56% among the elderly. The disparities associated with chronic pain go beyond age, with women, indigenous populations, and socioeconomically disadvantaged people being disproportionately affected [10–14]. Since some of these subgroups are underrepresented in traditional studies, the potential impacts of different treatments are largely unknown [10].

A significant challenge in understanding the full scope of chronic pain management is that a vast amount of relevant information is encapsulated within unstructured, free-text data. This includes detailed clinical narratives in electronic health records (EHRs) and patient-generated content on social media platforms, among others. EHRs, for instance, contain granular details about a patient’s subjective experience, past treatments, and the impact of pain on their life, which are often absent from structured data fields [15]. Similarly, social media have become valuable resources where patients discuss their lived experiences [16], share self-management strategies [17], and report on the effectiveness of various therapies, often with the goal of facilitating self-care [18]. A compounding challenge is that both chronic pain-related free-text data and associated literature are largely hidden within the vast amounts of non-chronic pain-related literature (e.g., acute pain). Consequently, obtaining relevant insights from the data and meta-review of the literature specific to chronic pain can be a complex endeavor.

In this challenging landscape, innovative technologies such as natural language processing (NLP) are emerging as promising tools to unlock these rich data sources [19]. As a branch of artificial intelligence, NLP has shown potential in various aspects of chronic pain management, with recent studies demonstrating its effectiveness in analyzing patient-reported outcomes, identifying imaging findings related to low back pain, and related topics [19, 20]. Despite the promise of NLP in chronic pain, the literature is dispersed. Therefore, a scoping review is necessary to consolidate existing knowledge, identify key advances and knowledge gaps in the literature, and inform future research directions in this emerging field. We conducted a scoping review of peer-reviewed studies published between 2014 and 2025. This period is marked by the introduction of Word2Vec [21], transformer models [22] such as BERT [23], and generative models like the GPT series [24].

The aim of this scoping review is to map the literature on the use of NLP, including recent LLMs, in chronic pain research to highlight the applications, methodological trends, and current limitations. In particular, we sought to answer the following questions:

(i) How is NLP used in chronic pain research, and what text data sources are analyzed?
(ii) What NLP methods are applied, and how have these methods evolved over time?
(iii) What limitations exist in current studies, and what future research directions are recommended?

## Methods

**Fig. 1:**
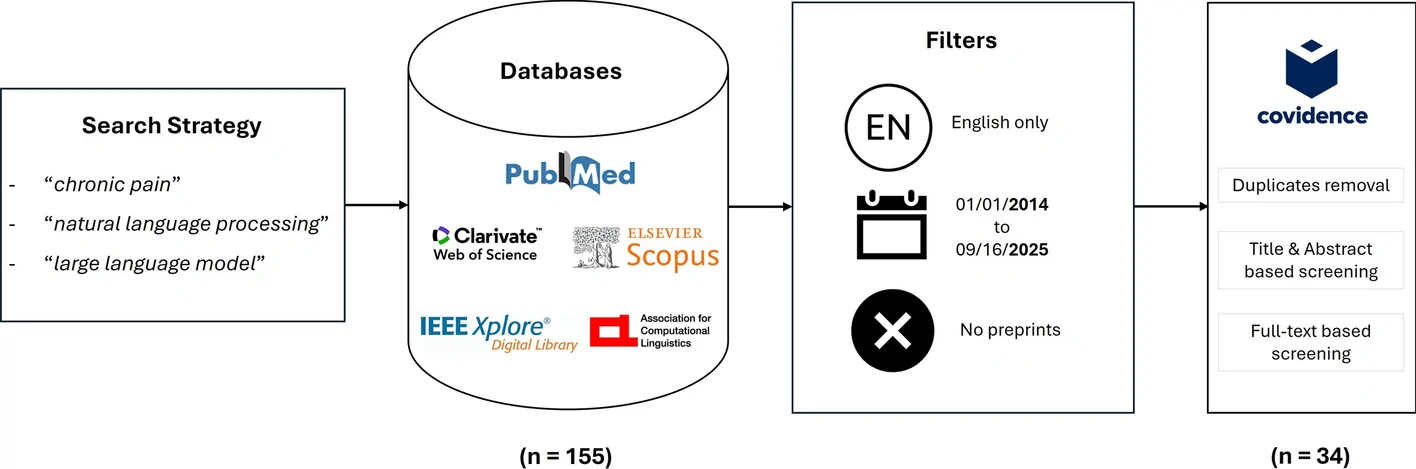
Workflow of literature identification and selection

This review is carried out under the PRISMA Scoping Review guidelines [25] and the checklist can be found in the supplementary file. The protocol was registered in Open Science Framework: 10.17605/OSF.IO/24ZYH. Figure 1 shows the literature selection workflow. Two reviewers (SR and AS) developed the search strategy and screened the titles and abstracts of all retrieved papers. Three papers were considered borderline and were discussed until agreement was reached on whether to include them. The full-text review, data extraction, and bias assessment were conducted by the primary reviewer (SR), with input from the senior authors (AS, SB) as needed. Formal inter-annotator agreement statistics were not recorded.

### Eligibility criteria

Eligible publications for this review were restricted to peer-reviewed published literature (observational studies, algorithm validation studies, computational model evaluations, experimental, qualitative), including journal articles and full conference papers. The study must be written in English, although the language of the textual dataset used may vary. The publication period of the included studies was restricted to the last decade, specifically from 2014 to 2025. To be included, a study should answer a research question(s) on the design, development, and application of NLP in chronic pain.

### Information sources

A search was conducted in the following databases from 1^st^ January 2014 until 16^th^ September 2025: PubMed, Web of Science, IEEE Xplore, Scopus, and ACL Anthology. The selected databases offer comprehensive coverage of both healthcare and NLP research. PubMed and Web of Science provide access to biomedical literature on chronic pain, while IEEE Xplore and ACL Anthology focus on technical advancements in NLP. Scopus bridges these disciplines, ensuring an interdisciplinary approach for a thorough review. Preprints (arxiv/biorxiv), forewords, prefaces, table of contents, programs, schedules, indexes, call for papers/participation, lists of reviewers, lists of tutorial abstracts, invited talks, appendices, session information, obituaries, book reviews, newsletters, lists of proceedings, lifetime achievement awards, erratum, systematic reviews, scoping reviews, and notes were excluded.

### Search strategy

We used three keywords: “chronic pain", “natural language processing", and “large language model". The search results are presented in Table 5 and strategies specific to each database are detailed below:

#### PubMed

(chronic pain) AND ((natural language processing) OR (large language model)); Filters: Adaptive Clinical Trial, Books and Documents, Case Reports, Classical Article, Clinical Conference, Clinical Study, Clinical Trial, Dataset, Meta-Analysis, Observational Study, Observational Study & Veterinary, Randomized Controlled Trial, English. These PubMed-specific filters (e.g., language and article type) were applied to improve search precision and limit the retrieval of non-relevant records such as editorials, commentaries, or non-English publications outside the scope of this review.

#### Web of science

"chronic pain (All Fields)" AND ("natural language processing" OR "large language model"); Filters: (All Fields) and English (Languages).

#### IEEE Xplore

("All Metadata": "chronic pain") AND ("All Metadata": "natural language processing" OR "All Metadata": "large language model"); Filters: 2014 - 2025.

#### Scopus

Full search query: TITLE-ABS-KEY (( "chronic pain" ) AND (( "natural language processing" ) OR ( "large language model" ))) AND ( LIMIT-TO ( DOCTYPE, "ar" ) OR LIMIT-TO ( DOCTYPE, "cp" )) AND ( LIMIT-TO ( LANGUAGE, "English" )) AND ( LIMIT-TO ( PUBSTAGE, "final" ))

#### ACL Anthology

The ACL Anthology does not currently provide functionality for batch exporting records. The following search query string was input in the search box: ("chronic pain") AND ("natural language processing" OR "large language model"). We used the Zotero extension to export results.

### Selection process

Each study was independently screened for eligibility by marking it as ‘yes’ (for inclusion), ‘no’ (for exclusion) or ‘maybe’ (in case of uncertainty about relevance) on the Covidence platform (https://app.covidence.org). Full access to the Covidence was provided via Emory University credentials. The search query results from each database were exported in RIS format (except for ACL anthology since it does not provide functionality to batch export records) and then imported into Covidence platform which removed all duplicates. In the first stage, we screened the titles and abstracts of each study as identified in the databases by the search strategies. In the second stage of screening, the full-text manuscripts were screened as per the eligibility criteria. Studies that did not meet the eligibility criteria were excluded.

### Data abstraction

We extracted data from the final included full-texts. Before formal extraction, the data extraction form was piloted with a sample paper to identify and address any issues in the form to ensure it was comprehensive. The data extraction was then conducted on all the included papers (i.e., 34).

### Data items

For each selected article, we extracted the following data items: year of publication, study design, research question, dataset description, NLP technology used, number of participants and their age range, primary results, and reproducibility characteristics (i.e., availability of code via GitHub, supplementary code, Zenodo, etc.).

### Study risk of bias assessment

Risk of bias refers to the potential for systematic errors within the design, conduct, or analysis of individual studies that may compromise the internal validity of findings. The risk of bias in the included studies was assessed using a simplified checklist inspired by the Joanna Briggs Institute (JBI) Critical Appraisal Tools [26]. The checklist was tailored to evaluate studies employing NLP techniques for tasks related to chronic pain. The checklist included 10 items such as study aim, relevance to chronic pain, NLP technique description, limitations, and others (see Table 1 for checklist). For each item, studies were rated as “Yes," “No", or “Unclear." A composite score was then calculated to classify the overall risk of bias into three categories: low (*≥ 8* ‘Yes’), moderate (5-7 ‘Yes’), or high (*≤ 5* ‘Yes’).

### Reporting bias assessment

Reporting bias refers to selective or incomplete disclosure of study findings, affecting transparency and reproducibility. To assess the risk of bias due to missing results in the synthesis, a customized checklist was developed, inspired by the ROBINS-I tool [27], a framework for evaluating potential bias in non-randomized studies across multiple methodological domains. This checklist was modified to evaluate reporting bias in studies employing NLP techniques for chronic pain and focused on the completeness of results, selective reporting, and transparency. Each study was assessed using this checklist (shown in Table 2), with responses recorded as “Yes," “No," or “Unclear." A composite score was calculated for each study to classify the risk of reporting bias as low risk (7–8 “Yes"), moderate risk (4–6 “Yes"), and high risk (<4 “Yes"). No statistical methods were applied to detect publication bias due to the heterogeneity of the included studies.

### Ethical considerations

This review involved the analysis of previously published work and did not require ethical approval or patient consent (IRB Exempt at Emory University).

## Results

### Study selection and characteristics

**Fig. 2:**
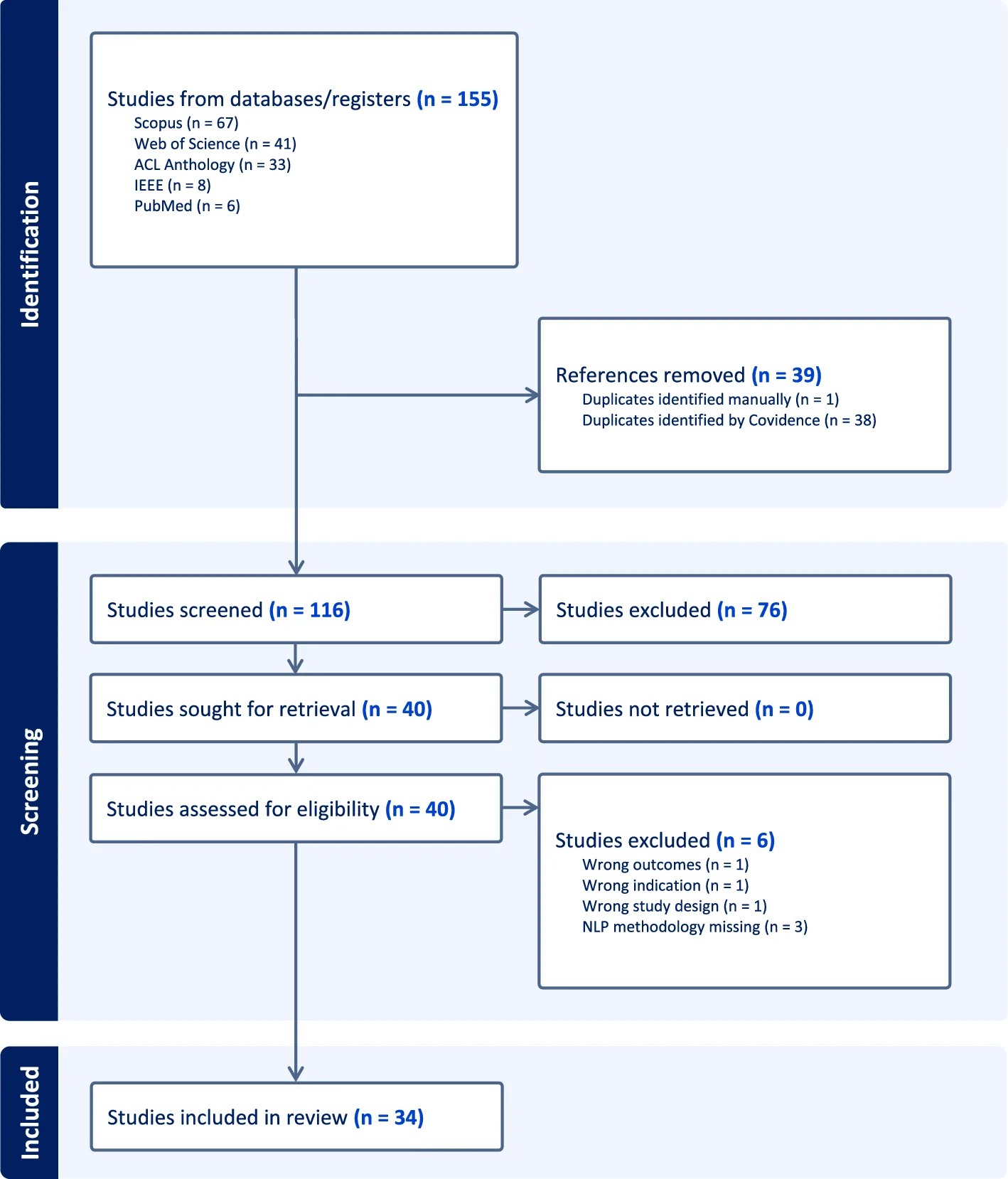
PRISMA flow diagram depicting the data collection and selection processes, and the number of articles at each step

**Fig. 3:**
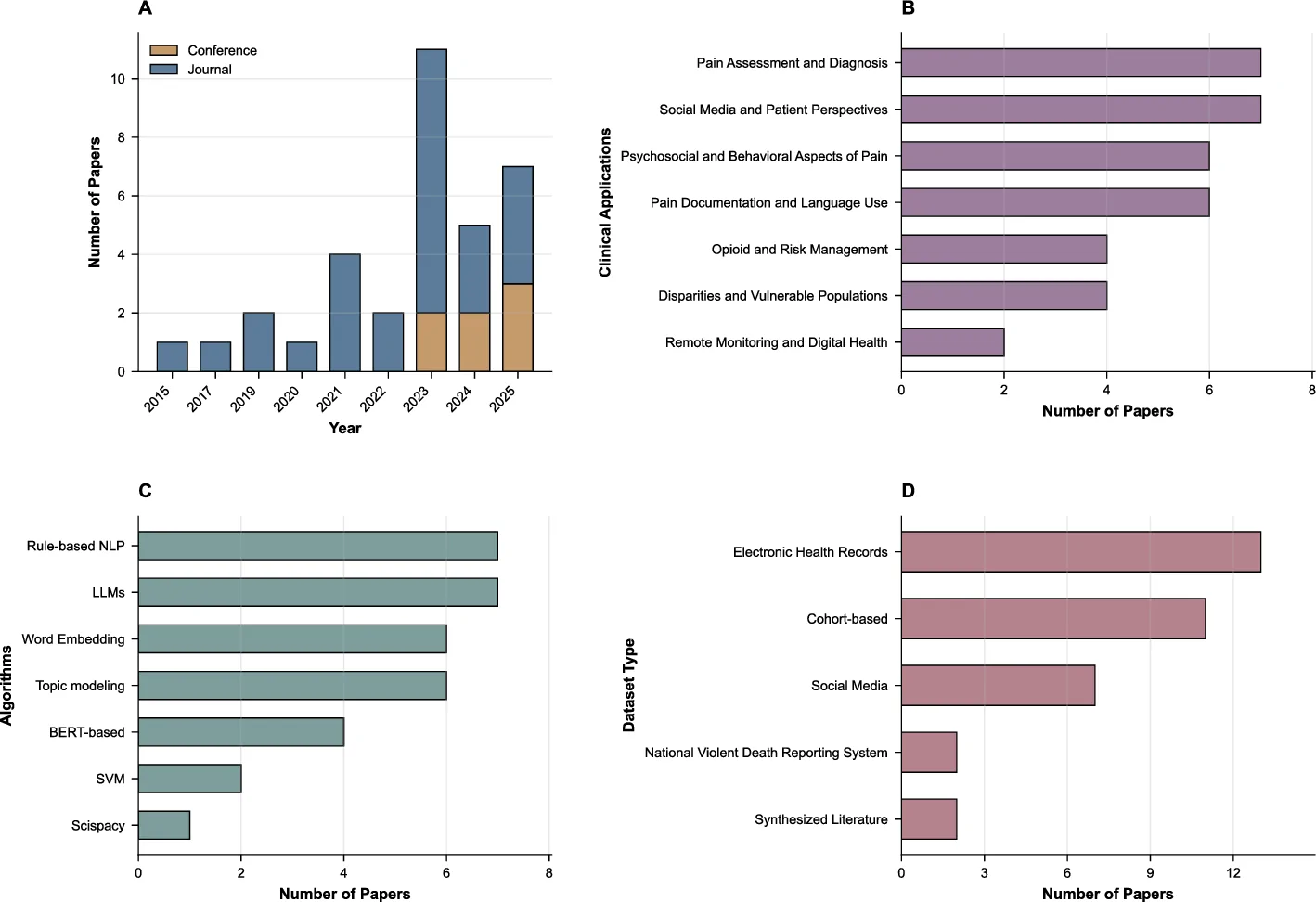
Characteristics of included studies. (A) Publications by year and venue type. (B) Potential clinical applications. (C) NLP algorithms used. (D) Dataset types. Multiple categories per study were counted separately

The study screening process is outlined in Fig. 2. Following title, abstract, and full-text screening, 34 studies were included in the final review. These studies were published between 2015 and 2025 (Fig. 3A). The majority of studies (23/34; 67%) were published in the past three years, highlighting the growing interest in applying NLP techniques to chronic pain data. Table 3 provides detailed data extraction results from individual studies. 11/34 papers (32%) are concentrated in five venues, including journals and conference proceedings: Pain, International Journal of Medical Informatics, JAMA Network Open, Journal of Medical Internet Research, and the IEEE International Conference on Digital Health (ICDH). The remaining 23 papers (68%) are spread one per venue across outlets such as BMJ Open, npj Digital Medicine, Journal of Pain Research, Journal of Biomedical Informatics, and others.

#### Clinical applications

Figure 3B shows several patterns of clinical applications that emerged in the research questions of the papers in this review. For instance, some studies investigated whether clinical or patient data could be used to predict outcomes, such as opioid misuse or worsening mental health [28–33]. Six focused on how people talk about pain, such as how patients describe it or how healthcare providers use language that might carry stigma [34–39]. Multiple studies explored what people with chronic pain share over social media [18, 40–44]. Five studies investigated treatment responses, including what might influence whether a treatment or placebo works [34, 45–48]. Another five examined how pain connects with mental health conditions like depression or suicide [30, 33, 49–51]. Six studies focused on how people experience or understand their pain in daily life [35, 38, 39, 52–54]. One study focused on building a new dataset to support future work [55]. These applications reflect a diversity of goals: some aim to improve care through data, while others seek to better understand how people live with and talk about chronic pain.

In terms of NLP methodology, information extraction and named entity recognition methods featured prominently. Studies involving such methods primarily focused on extracting pain-related concepts and clinical risk indicators from healthcare text. Key target concepts included pain mentions with anatomical locations, pain characteristics (chronic/acute), and pain management measures from mental health records [31, 56], opioid agreement violations and problematic use behaviors from clinical notes [28, 36], and complementary therapy usage patterns [50]. Additional extraction targets included patient experience dimensions using structured frameworks like International Classification of Functioning, Disability, and Health (ICF) with aspect-based sentiment analysis [38] and pain care quality indicators [30].

#### Dataset

As shown in Fig. 3D, electronic health records (EHRs) were the most frequent source of data [28–32, 36, 37, 44, 46, 50, 52, 56], spanning structured hospital admission notes [37], the MIMIC-III dataset [29, 44], CRIS mental health records and linked Lambeth primary care data [31, 56], the VHA Corporate Data Warehouse [30, 46, 50], Vanderbilt’s BioVU biobank [52], and the Group Health Cooperative [28]. Social media data appeared in 7/34 (21%) [18, 40–44, 48], primarily sourced from Reddit (e.g., [40–42]) and Twitter (X) (e.g., [18, 48]). Interview-based or longitudinal cohort datasets were used in 11/34; 32% [30, 35, 38, 39, 45–47, 50, 52–54], including survey-based studies, patient interviews, and wearable-data-linked tracking platforms. The U.S. National Violent Death Reporting System (NVDRS) appeared in 2/34; 6% [49, 51]. Two studies (2/34; 6%) synthesized published literature rather than analyzing patient-level data [55, 57].

#### Methodology

**Fig. 4:**
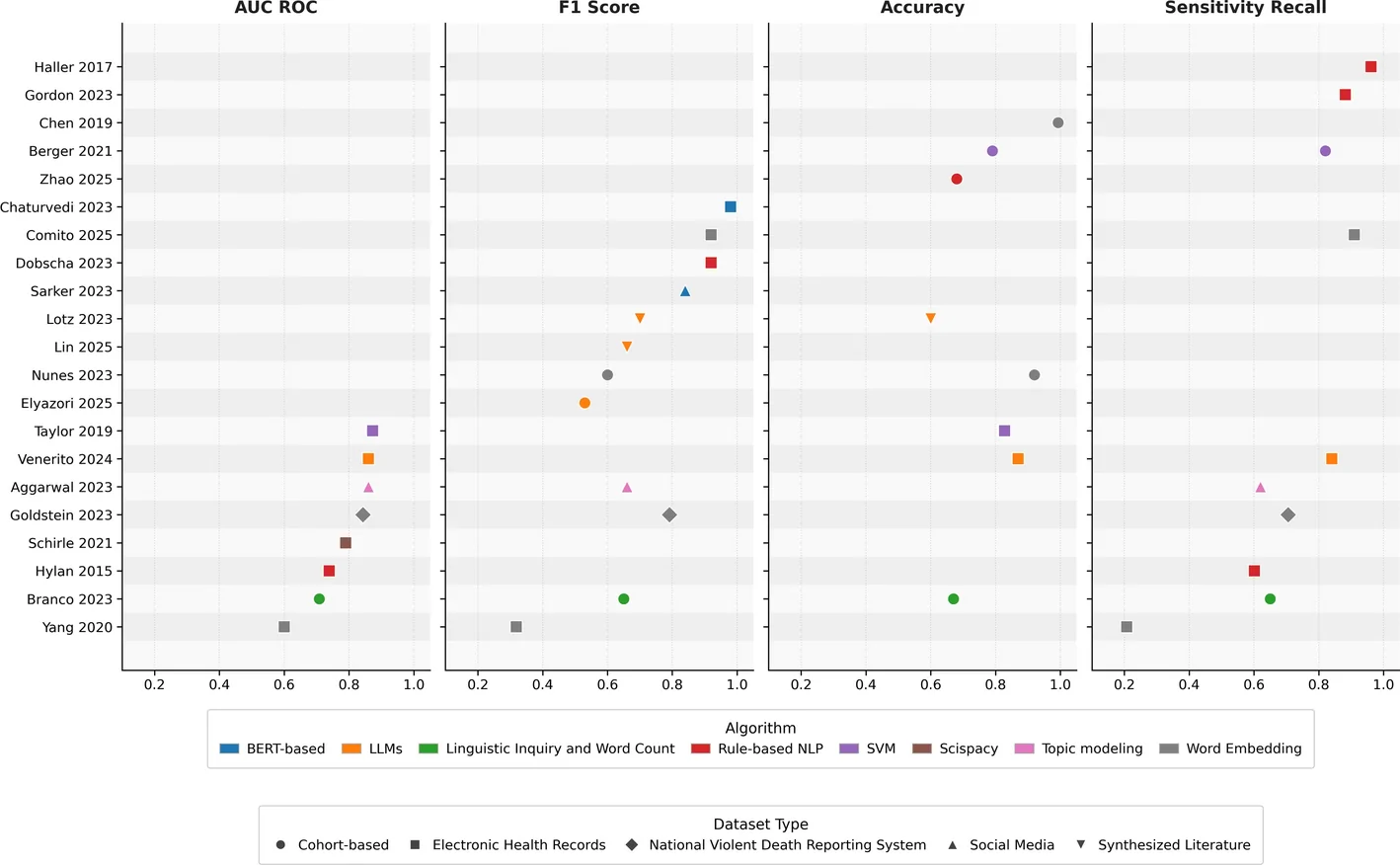
AUC ROC, F_1_ score, accuracy, and sensitivity/recall across studies reporting these metrics, stratified by algorithm type and dataset source. Subset of included studies is shown, as remaining studies reported alternative performance measures

A wide range of NLP methods were used across the 34 studies (Fig. 3C), with a clear shift toward more advanced models over time. Early studies used rule-based approaches such as regular expressions [30, 33, 34, 43], though it is often used in combination with other techniques such as classical machine learning models like SVMs and logistic regression [29, 45–47, 50, 51, 53, 56]. Topic modeling with LDA was used in a number of studies [35, 40, 41, 43, 48, 49, 58]. Embedding-based methods such as Word2Vec and FastText were seen in 8 studies [18, 29, 31, 37, 43, 44, 53, 56]. Notably, studies from 2023 onward used transformer-based models, including BERT, BioBERT, RoBERTa, Longformer, and GPT-based models [18, 31, 37–39, 43, 53–57, 59]. This reflects a growing trend in applying large language models (LLMs) to understand pain, mental health, and patient communication in recent work. Figure 4 further summarizes the reported evaluation metrics across studies, showing AUC-ROC, F1 score, accuracy, and sensitivity/recall stratified by algorithm type and dataset source.

### Risk of bias in studies and reporting bias

**Table 1:**
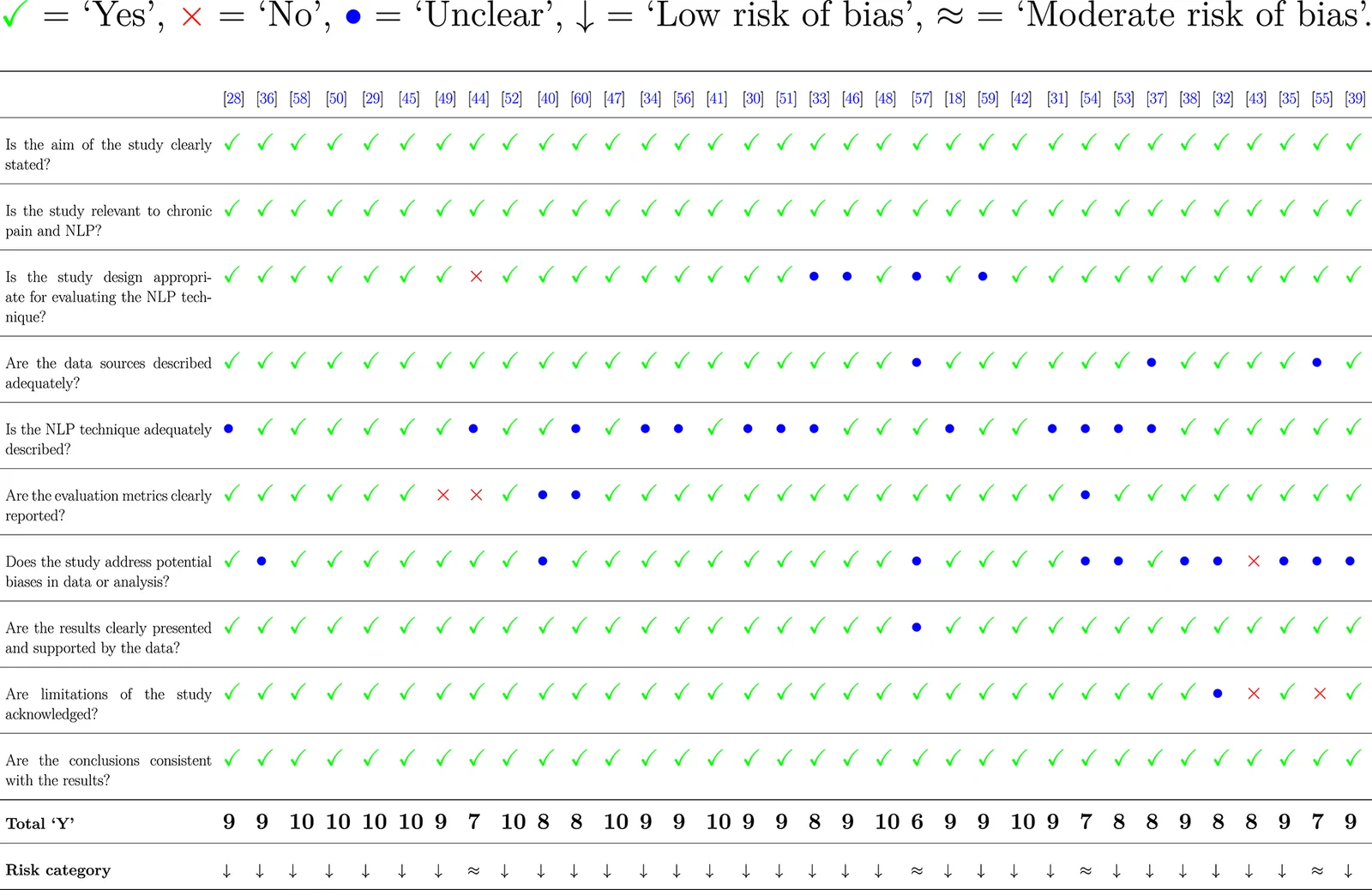
Risk of bias assessment for included studies

* \checkmark * = ‘Yes’, *× * = ‘No’, *∙ * = ‘Unclear’, *↓ * = ‘Low risk of bias’, *≈ * = ‘Moderate risk of bias’

The risk of bias was assessed for all 34 included studies using a simplified checklist as discussed in Sect. 2.7. The results of the risk of bias assessment are summarized in Table 1. Of all studies, 30 studies fall under low, 4 under moderate, and 0 under high risk of bias. Overall, most included studies demonstrated a low risk of bias, indicating that their findings can be considered reliable. A small number of studies showed moderate concerns, but none were judged to be at high risk of bias. Table 2 presents results of the risk-of-bias assessment specific to missing results and reporting bias. A total of 13 studies were classified as low risk, 21 as moderate risk, and 0 as high risk. Several plausible biases were found for the studies included in this literature review, such as the lack of preregistration for the study or the reference to a preregistered protocol. This shows a gap in ensuring transparency in research design and execution. Furthermore, some studies did not provide access to supplementary materials, which limited the replicability and verification of reported findings. These findings emphasize the importance of adhering to open science practices to mitigate risks of reporting bias. The findings also highlight variability in reporting practices and the need for consistent adherence to robust reporting standards in NLP-based chronic pain research.

**Table 2:** Risk of reporting bias and missing results

	[28]	[36]	[58]	[50]	[29]	[45]	[49]	[44]	[52]	[40]	[60]	[47]	[34]	[56]	[41]	[30]	[51]
Are all datasets described in the methods section also reported in the results section?	*\checkmark *	*\checkmark *	*\checkmark *	*\checkmark *	*\checkmark *	*\checkmark *	*\checkmark *	*\checkmark *	*\checkmark *	*\checkmark *	*\checkmark *	*\checkmark *	*\checkmark *	*\checkmark *	*\checkmark *	*\checkmark *	*\checkmark *
Are all evaluation metrics listed in the methods section reported in the results?	*\checkmark *	*\checkmark *	*\checkmark *	*× *	*\checkmark *	*× *	*× *	*× *	*\checkmark *	*\checkmark *	*∙ *	*\checkmark *	*\checkmark *	*× *	*× *	*× *	*\checkmark *
Does the study report results for all intended tasks or objectives?	*\checkmark *	*\checkmark *	*\checkmark *	*\checkmark *	*\checkmark *	*\checkmark *	*\checkmark *	*\checkmark *	*\checkmark *	*\checkmark *	*\checkmark *	*× *	*\checkmark *	*× *	*\checkmark *	*\checkmark *	*\checkmark *
Are the results for negative or null findings explicitly reported?	*\checkmark *	*\checkmark *	*\checkmark *	*∙ *	*× *	*\checkmark *	*\checkmark *	*\checkmark *	*\checkmark *	*\checkmark *	*\checkmark *	*\checkmark *	*\checkmark *	*\checkmark *	*\checkmark *	*\checkmark *	*\checkmark *
Is there consistency between the study’s objectives and its results?	*\checkmark *	*\checkmark *	*\checkmark *	*\checkmark *	*\checkmark *	*\checkmark *	*\checkmark *	*\checkmark *	*\checkmark *	*\checkmark *	*\checkmark *	*\checkmark *	*\checkmark *	*\checkmark *	*\checkmark *	*\checkmark *	*\checkmark *
Does the study acknowledge any missing results or limitations in reporting?	*\checkmark *	*\checkmark *	*\checkmark *	*\checkmark *	*\checkmark *	*\checkmark *	*\checkmark *	*\checkmark *	*\checkmark *	*\checkmark *	*\checkmark *	*\checkmark *	*\checkmark *	*\checkmark *	*∙ *	*\checkmark *	*\checkmark *
Was the study pre-registered, or does it reference a pre-registered protocol?	*× *	*× *	*∙ *	*× *	*× *	*\checkmark *	*× *	*× *	*× *	*× *	*× *	*\checkmark *	*\checkmark *	*× *	*× *	*× *	*× *
Does the study provide access to supplementary material or raw data?	*× *	*\checkmark *	*× *	*× *	*× *	*\checkmark *	*× *	*\checkmark *	*\checkmark *	*∙ *	*\checkmark *	*\checkmark *	*∙ *	*\checkmark *	*∙ *	*\checkmark *	*\checkmark *
**Total ‘Y’**	**6**	**7**	**6**	**4**	**5**	**7**	**5**	**6**	**7**	**6**	**6**	**7**	**7**	**5**	**4**	**6**	**7**
**Risk category**	*≈ *	*↓ *	*≈ *	*≈ *	*≈ *	*↓ *	*≈ *	*≈ *	*↓ *	*≈ *	*≈ *	*↓ *	*↓ *	*≈ *	*≈ *	*≈ *	*↓ *

	[33]	[46]	[48]	[57]	[18]	[59]	[42]	[31]	[54]	[53]	[37]	[38]	[32]	[43]	[35]	[55]	[39]
Are all datasets described in the methods section also reported in the results section?	*\checkmark *	*\checkmark *	*\checkmark *	*\checkmark *	*\checkmark *	*\checkmark *	*\checkmark *	*× *	*\checkmark *	*\checkmark *	*\checkmark *	*\checkmark *	*\checkmark *	*\checkmark *	*\checkmark *	*\checkmark *	*\checkmark *
Are all evaluation metrics listed in the methods section reported in the results?	*\checkmark *	*\checkmark *	*\checkmark *	*× *	*× *	*\checkmark *	*\checkmark *	*× *	*\checkmark *	*\checkmark *	*\checkmark *	*\checkmark *	*\checkmark *	*\checkmark *	*\checkmark *	*\checkmark *	*\checkmark *
Does the study report results for all intended tasks or objectives?	*\checkmark *	*\checkmark *	*\checkmark *	*∙ *	*× *	*\checkmark *	*\checkmark *	*\checkmark *	*\checkmark *	*\checkmark *	*\checkmark *	*\checkmark *	*\checkmark *	*\checkmark *	*\checkmark *	*\checkmark *	*\checkmark *
Are the results for negative or null findings explicitly reported?	*\checkmark *	*\checkmark *	*\checkmark *	*∙ *	*\checkmark *	*\checkmark *	*\checkmark *	*\checkmark *	*\checkmark *	*\checkmark *	*\checkmark *	*\checkmark *	*\checkmark *	*× *	*\checkmark *	*\checkmark *	*\checkmark *
Is there consistency between the study’s objectives and its results?	*\checkmark *	*\checkmark *	*\checkmark *	*\checkmark *	*\checkmark *	*\checkmark *	*\checkmark *	*\checkmark *	*\checkmark *	*\checkmark *	*\checkmark *	*\checkmark *	*\checkmark *	*\checkmark *	*\checkmark *	*\checkmark *	*\checkmark *
Does the study acknowledge any missing results or limitations in reporting?	*\checkmark *	*\checkmark *	*\checkmark *	*\checkmark *	*\checkmark *	*\checkmark *	*∙ *	*\checkmark *	*\checkmark *	*\checkmark *	*\checkmark *	*\checkmark *	*\checkmark *	*\checkmark *	*\checkmark *	*∙ *	*\checkmark *
Was the study pre-registered, or does it reference a pre-registered protocol?	*× *	*× *	*× *	*× *	*× *	*× *	*× *	*× *	*\checkmark *	*\checkmark *	*× *	*× *	*× *	*× *	*× *	*× *	*× *
Does the study provide access to supplementary material or raw data?	*× *	*× *	*\checkmark *	*\checkmark *	*\checkmark *	*\checkmark *	*× *	*∙ *	*× *	*× *	*× *	*× *	*\checkmark *	*× *	*\checkmark *	*× *	*\checkmark *
**Total ‘Y’**	**6**	**6**	**7**	**4**	**5**	**7**	**5**	**4**	**7**	**7**	**6**	**6**	**7**	**5**	**7**	**5**	**7**
**Risk category**	*≈ *	*≈ *	*↓ *	*≈ *	*≈ *	*↓ *	*≈ *	*≈ *	*↓ *	*↓ *	*≈ *	*≈ *	*↓ *	*≈ *	*↓ *	*≈ *	*↓ *

*\checkmark * = ‘Yes’, *× * = ‘No’, *∙ * = ‘Unclear’, *↓ * = ‘Low risk of bias’, *≈ * = ‘Moderate risk of bias’

**Table 3:** Summary of the 34 included Studies

YearRef.	Study Design	Research Question	Dataset	NLP Tech.	Participant Count	Age (Years)	Results
2015 [28]	Observational, longitudinal	Can EHR data predict problem opioid use in chronic opioid therapy?	2,752 noncancer pain patients from Group Health Cooperative (2008–2010)	Regular Expression	2,752 (train: 1,449 & Val: 1,303)	18+	C-statistic: 0.739 (95% CI = .688, .790); Sensitivity: 60.1%; Specificity: 71.6%; PPV: 11.4%; NPV: 96.7%
2017 [36]	Retrospective cohort	Automated risk assessment for chronic pain patients using NLP and EHR data to populate the Opioid Risk Tool	Essentia Health Epic EHR system (2007-2012)	Regular expression	3668 patients	Median 48 years (25th-75th percentile: 37-58 years)	NLP achieved 96.1% sensitivity, 92.8% specificity, 92.6% PPV for detecting violations. High-risk patients (ORT 8+) had 3x higher violation rates.
2019 [58]	Observational, Secondary data analysis	How text mining & visual analytics reveal engagement in adolescent pain interventions?	123 adolescents with chronic idiopathic pain & their parents in an internet-based CBT intervention	LDA,k-means clustering	273	11-17	Participants’ messages acc: 76.5%, Coaches’ messages acc: 99.3%
2019 [50]	retrospective cohort, observational	What are the frequency and predictors of CIH therapy use in veterans with chronic pain?	Retrospective cohort of 530,216 younger veterans with chronic MSK pain (2010–2013, VHA)	SVM	530,216	18-40	AUC ranged from 82.8% to 91.8%, Accuracy ranged from 79.8% to 85.8%
2020 [29]	Observational, retrospective	How can DL & NLP improve disorder classification from discharge summaries?	MIMIC-III	Word and sentence-level CNN, SVM with id-itf & word embedding	1610	N/A	The averaged metrics are Precision: 82.30%, Recall: 20.72%, ROCAUC: 60.00%, and F1: 31.89%
2021 [45]	Randomized, double-blind, placebo-controlled	Can patient discourse features identify placebo responders in back pain?	Exit interviews from 66 chronic back pain patients (NCT02013427)	Logistic Regression, Linear SVM, L1 (lasso) regularization, L2 (ridge) regularization	125	18+Men Avg.: 48.8±1.9; Women Avg. 41.84±2.7	Accuracy: 79%, Sensitivity: 0.82, Specificity: 0.75 (SVM with L1 regularization)
2021 [49]	Retrospective	What job issues do nurses face before suicide?	NVDRS (2003–2017, CDC) data on 203 nurse suicides	LDA, Treebank Word Tokenizer, WordNet Lemmatizer	203 nurses	21+	N/A
2021 [44]	Observational, exploratory analysis	How is pain described, & which texts aid in creating a pain lexicon?	CRIS, MIMIC-III, Twitter (‘chronic pain’ tweets), Reddit (chronic pain subreddit)	Word2Vec, FastText	200 documents(50 per dataset)	N/A	N/A
2021 [52]	Retrospective observational pilot	How can data-driven methods in EHRs identify problematic opioid use?	Vanderbilt’s BioVU biorepository	Text-based Score using ScispaCy	29868	20+	AUC for Text-based score: 0.79, AUC for Comorbidity score: 0.76
2022 [40]	Observational	What do chronic pain patients discuss on Reddit?	937 text posts from ‘r/ChronicPain’ subreddit (collected via PRAW on 6 Sept 2021)	LDA, Bag-of-words model	709 unique authors	N/A	N/A
2022 [60]	cross-sectional observational	How often does stigmatizing language in admission notes vary by condition and race?	48651 admission notes (2018) from a large urban medical center	Tokenization into unigrams & bigrams, Porter2 stemming algorithm	29,783 patients, 1,932 clinicians	Mean: 46.9SD: 27.6	N/A
2023 [47]	Double-blind, placebo-controlled	Can language predict placebo response and distinguish drug from placebo responders?	Two RCTs (NCT02013427) on chronic back pain w/ language interviews	Linguistic Inquiry Word Count, Semantic Proximity	Study 1: 66, Study 2: 50	Mean: 45.3, SD: 2.3	Placebo arm: AUC 0.708, F1 0.65, Precision 0.68, Recall 0.65, Balanced Acc. 67%
2023 [34]	randomized, placebo-controlled	Does reattributing back pain to mind/brain processes relieve pain in PRT?	Chronic back pain adults responses on pain causes (NCT03294148, 2017–2019)	Text scaling (LSA/PCA), Rulebased (keywords matching)	151	21-70	Cohen *κ * at:pretreatment: 0.42, posttreatment: 0.68
2023 [56]	Observational, retrospective	How can we create a labeled pain corpus from mental health EHRs for NLP?	CRISA manually annotated corpus of pain mentions in mental health EHRs	KNN, SVM, BERT	723 patients	*≤ *20: 10%, 21-40: 21%, 41-60: 25%, 61-80: 33%, >80: 10%	F_1_: 0.98(95% CI 0.98-0.99)
2023 [41]	Observational	How can the RRCP dataset model chronic pain experiences on Reddit?	Reddit Reports of Chronic Pain (RRCP)86537 submissions (2013–2020) from 12 chronic pain-related subreddits	K-Means, LDA, VADER sentiment analysis	44,815 unique authors	13+	N/A
2023 [30]	retrospective cohort, observational	Are mental health conditions linked to pain care quality in VHA primary care?	VHA CDW linked to MSD cohort w/ EHR data from 134,508 visits with moderate/severe MSK pain (2017)	Rule-based NLP	62,721 Veterans	Avg.: 50.5 yrs, SD: 16.6	F-measure: 0.92
2023 [51]	Observational, retrospective	What circumstances precede female firearm suicide in coroner & police reports?	NVDRS (2014–2018) data on female firearm suicides (40 states, Puerto Rico)	Naive Bayes, Random Forest, SVM, Gradient Boosting, Lemmatization, TF-IDF	1462	Mean: 47, SD: 6.9	SVM: Specificity 98.1%, Sensitivity 70.5%, F_1_ 79.1, PPV 90.5%, AUC-ROC GB: Specificity 98.7%, PPV 93.2%.
2023 [33]	Retrospective cross-sectional	Do pain screening and reports differ between LGBT and non-LGBT Veterans?	VHA CDW (FY 2010–2019), covering over 9 million Veterans	Keywords based matching (Regex)	1,149,486 (LGBT: 218,154)	10-year age groups: <25 to *≥ *75	Sensitivity: 88.2%,Specificity: 91.5%
2023 [46]	Observational, single-site, proof-of-concept	How can NLP estimate pain intensity from patient narratives?	Interviews with chronic pain patients from Centro Hospitalar Universitário de São João (Oct 2019–Oct 2020)	TF-IDF, POS TF-IDF, SVM, DT	94	Avg: 56.4 ± 12.7	SVM F_1_: 0.60, Classification acc.:92% mild, 42% moderate, 13% severe
2023 [48]	Observational, retrospective, stratified analysis	Does Twitter language predict community pain and its variations beyond demographics?	Gallup-Sharecare Well-Being IndexCTLB (*∼ *1.5B county-level tweets)*∼ *1.8M pain-related tweets matched to Gallup counties	Extra Tree Classifier, RF, Content Specific LDA	2,541,688 responses	N/A	RF: AUC 0.86, F_1_ 0.64, Precision 0.85, Recall 0.61, ETC: AUC 0.86, F_1_ 0.66, Precision 0.84, Recall 0.62
2023 [57]	exploratory methodological	How can LLMs, SGs, & KGs synthesize literature to aid chronic back pain research?	83 LBP literature	GPT3 & GPT4, SGs, KGs	N/A	N/A	Acc: 60%; F_1_: 0.70 (biomechanics), 0.70 (psychology), 0.36 (both), 0.46 (neither)
2023 [18]	Observational study	How can Twitter reveal chronic pain experiences & alternative therapies?	4,998 annotated tweets with chronic pain hashtags/keywords, labeled for self-reported chronic pain experiences	RoBERTa, SciBERT, BioClinicalBERT, BERTweetBioBERT, Clinical_KB_BERT	22,795 Twitter users	N/A	RoBERTa F_1_: 0.84(95% CI: 0.80–0.89)
2024 [59]	retrospective observational	Can LLM sentiment analysis aid fibromyalgia (FM) diagnosis via pain patterns?	Patients with and without FM (per 2016 ACR criteria) chronic pain from a rheumatology clinic (Jan 8–20, 2024).	Mistral-7B-Instruct-v0.2, HuggingFace Transformers API for attention weight analysis	80 (FM: 40, non-FM pain controls: 40)	*∼ *38.76 to 57.68	Prompt-engineered:Acc. 0.87, Precision 0.92, Recall 0.84, Specificity 0.82, AUROC 0.86
2024 [42]	Observational, retrospective	Do online peer support users align their pain language with others?	Reddit users’ comment histories in a chronic pain support community (10 years, 68,206 interactions) with *≥ *100 posts per user	Linguistic Inquiry and Word Count (LIWC) dictionary, VADER	199 users	N/A	t(198)=4.02, *p<.001*, d=0.40 (common power)
2024 [31]	observational, cross-sectional, retrospective	What are the demographics and diagnoses of pain in mental health records, and how do they overlap in care?	CRISLambeth DataNet (primary care records), linked for patient-level analysis across care settings	SapBERT, MedCATTrainer for NER	27211	18+Mean: 44IQR: 29-55	F_1_ for:Pain classification: 0.98, Anatomy identification: 0.94
2024 [54]	longitudinal, observational	How can sentiment analysis track health in chronic pain patients?	ENVISION Study (NCT03240588) on chronic back/leg pain: 34,447 free-text samples (Nov 2019–2023)	Sentence Transformers, Watson NLU, k-means clustering, Hugging Face hub Transformers	242	N/A	N/A
2024 [53]	Prospective, observational	How can text and audio from chronic pain patients infer well-being?	Text & voice responses from chronic pain patients in NAVITAS & ENVISION studies (Dec 2019–July 2023, 30 U.S. sites)	RoBERTa, GeMAPS v2.0, Whisper OpenAI, Lasso regression, Ridge regression, SVM	166	Text responses: 49.6-73.2; Voice responses: 52-74	*ρ *: Mood (text) r=0.46, Pain (text) r=0.44, Sleep (text) r=0.40, Pain (voice) r=0.45, Alertness (voice) r=0.42
2025 [37]	Retrospective, multi-year	Can NLP classify chronic pain cases from short, fragmented clinical documentation (in Italian)?	Real-world medical and nursing clinical notes from Perugia Hospital	XGBoost, Gradient Boosting (GBM), BioBit, bert-base-italian-xxl-uncased, TF-IDF, Word2Vec, FastText	727 (179 from chronic pain notes + 548 from non chronic pain notes)	The study intentionally excludes age for ethical and methodological reasons.	(Best) XGBoost + TF-IDF = F_1_: 0.92 ± 0.01, Precision: 94%, Recall: 91%, Specificity: 93%
2025 [38]	Qualitative, exploratory	Can patient experiences of chronic pain (in semi-structured interviews) be analyzed using NLP and LLMs to support patient-centered assessment strategies?	Transcripts from semi-structured interviews (30–60 min each) w/ adults experiencing chronic pain.	LLMs (Meta Llama family, GPT-4, Llama-3.3-70B)	11	Mean age: 29.5 years (± 11.52)	LLM extraction performance (Llama-3.3-70B): F1 = 0.31 for ICF categories. F1 = 0.53 for sentiment labels.
2025 [32]	Experimental	Enhance RAG with knowledge graph-elicited reasoning for healthcare copilot diagnostic decision support	DDXPlus (public data, 49 diagnoses) and Chronic Pain Diagnostic Dataset (CPDD, private data from Tan Tock Seng Hospital, 33 diagnoses)	Knowledge graph-elicited reasoning RAG with four-tier hierarchical diagnostic knowledge graph, regular-expression NLP	DDXPlus: 1.3M patients (13,230 used); CPDD: 551 patients	N/A	MedRAG outperformed baselines with best L3 accuracy (66.04% CPDD, 68.01% DDXPlus). High-risk patients 3x more likely to have negative outcomes.
2025 [43]	Observational	explore chronic pain patterns, management approaches, and unmet needs among individuals with Systemic Lupus Erythematosus using Reddit discussions as a real-world data source	Reddit posts from r/Lupus and r/LupusSupport subreddits (2005-2024), over 30,000 posts	LLMs, regex-based lexicon matching, BERTopic for topic modeling	30,000 posts; 694 SLE users, 80 UCTD/MCTD users, 62 advice-seeking	N/A	Cannabis-related topics dominated discourse (342 forms, 116 general, 77 cannabinoids, 48 supplements). Focus on alternative therapies and care barriers.
2025 [35]	Cross-sectional analysis	Relationship between self-reported well-being practices and pain outcomes (pain interference and pain medication use) in adults with chronic pain using NLP	Midlife in US (MIDUS) study - open-ended survey responses from MIDUS 2, MIDUS Refresher, and MIDUS Milwaukee Refresher (2004-2015)	Network topic modeling using EGAnet package, Walktrap community detection algorithm, unigram tokenization, stemming	683 CP adults (373 MIDUS 2, 271 Refresher, 39 Milwaukee Refresher)	Mean 54.84 years (SD=12.30)	12 topics identified. Social connections reduced pain interference (*β *=-0.14, *p<.05*) and medication use (*β *=-0.11, *p<.05*). Topics captured unique well-being practices.
2025 [55]	Experimental	Impact of sentence context on zero-shot entity resolution accuracy in chronic lower back pain literature	Entity resolution dataset from Anderson et al. (2024) w/	GPT-3.5, spaCy for noun chunking and tokenization	501 entity pairs from CBP literature, labeled by 4 humans	N/A	Best F_1_ 0.66 (combined method) vs 0.63 (entity-only) vs 0.34 (full context). Strategic context integration superior.
2025 [39]	Experimental	Applying GPT-4 to assess pain severity and disability in patient written narratives	Fibromyalgia narratives (Serrat et al., 2022) plus FIQR, HADS, Tampa Scale responses	GPT-4 scored pain severity and disability (0-10 scales) at temperatures 0 and 1 with explanations	43 people with fibromyalgia	18+	GPT-4 achieved *>0.95* weighted agreement with experts, 0.43-0.49 correlations with FIQR, RMSE 1.20 (severity) and 1.44 (disability). Explanations rated adequate and clinically useful.

## Discussion

### Central findings

Based on the results presented in Sect. 3, it is evident that the use of NLP techniques for chronic pain research has evolved significantly over time, targeting an expansive range of topics and data sources. Early studies (e.g., 2015, 2019) often relied on rule-based methods like regular expressions and machine learning models such as support vector machines (SVMs), while more recent research has shifted toward advanced text modeling techniques such as transformers, including BERT [23], RoBERTa [61] and LLMs [38, 53, 57, 59]. This evolution reflects a growing sophistication in the field that is leveraging larger and lexically diverse datasets, such as MIMIC-III [62] and VHA, and social media based sources like Reddit and Twitter (X) to gain deeper insights into patient experiences.

The findings across studies reveal promising results in using automated and computational methods for health-related data analysis. For example, the use of NLP in [28] allowed for improved opioid use surveillance, demonstrating the potential of data-driven solutions to address specific health crises. Another key insight is the effectiveness of machine learning models, such as the ws-CNN used in [29], which enhances classification accuracy in patient phenotyping. Despite the different types of intervention, a recurring outcome is the growing evidence supporting automated methods for healthcare, highlighting increased accuracy and predictive capacity as consistent themes in the articles.

### Use of large language models

[57] explored GPT-3 for synthesizing chronic back pain literature, categorizing findings across biomechanics and psychology, though modest F_1_ (0.46 for combined domains) revealed challenges in interdisciplinary understanding. [59] used prompt-engineered sentiment analysis with Mistral-7B for fibromyalgia diagnosis, achieving high accuracy (0.87) but limited to a single application. [38] applied multiple LLMs including the Meta LLaMA family and GPT-4. [43] combined LLMs with regex-based lexicon matching and BERTopic for Reddit-based chronic pain analysis. [55] used GPT−3.5 for zero-shot entity resolution alongside spaCy for token labeling. [39] implemented GPT-4 (gpt-4-0125-preview) with temperature parameter adjustments for empathy detection.

Overall, 7/34 studies in this review utilized LLMs and all were published within the 2023–2025 period, pointing to very recent uptake. Despite their limited number, these studies represent diverse applications such as literature synthesis, diagnostic classification, multimodal monitoring, and qualitative data extraction. This pattern suggests that LLMs are gaining traction as complementary tools in chronic pain research. Early results highlight both their promise and the need for methodological refinement before widespread clinical adoption.

### Potential implications for clinical practice

While many studies in this review focused primarily on methodological development or exploratory analysis, a subset explicitly discussed potential clinical implementation. Specifically, nine studies described applications that could directly support clinical workflows, such as automated risk assessment using EHR text, prediction of opioid misuse, monitoring patient-reported outcomes, or assisting diagnostic decision support systems. For example, works such as [32, 34, 36, 39, 44, 47, 53, 54, 59] frame their NLP approaches as tools that could augment clinical decision-making, patient monitoring, or diagnostic support, indicating a translational orientation beyond purely exploratory research. These findings show that NLP tools (including LLMs) have the potential to support everyday pain care in practical ways. They can help clinicians notice early signs of risk, such as changes in mood or unsafe medication patterns, by scanning patient notes and other text. This may allow earlier follow-up and safer care. Such technology can also reveal key details from patient stories, which may help clinicians understand what factors matter most to patients, not just what is in a pain score, and support more personalized care. At the same time, NLP tools are not perfect. They may exhibit systematic biases or underperform for some patient groups. Consequently, NLP and LLMs, in their current form, should involve humans in the loop. To summarize, NLP has the potential to serve as an important toolset to inform care providers, aid them in understanding patient needs more comprehensively, and assist them in addressing patient needs faster, as long as the tools are used with care and oversight.

### Research gap

#### Methodological rigor of NLP techniques

The reviewed studies show variation in how NLP methods were implemented. Earlier studies primarily used rule-based approaches, topic modeling, or classical machine learning models, while more recent work has adopted transformer models and LLMs (Figs. 3C and 4). However, most LLM-based studies relied on prompting rather than model training. One study used few-shot learning with AI chaining [57], one applied prompt engineering [59], and four relied on zero-shot prompting [38, 39, 43, 55]. Another study used the Whisper API primarily for transcription before extracting textual features for analysis [53]. Retrieval-augmented generation (RAG) frameworks were rarely explored, with only one study explicitly implementing this approach [32]. The limited use of fine-tuning, retrieval-based methods, and systematic comparisons between modern LLMs and traditional models makes it difficult to assess the true benefits of newer techniques. Future studies should therefore emphasize stronger methodological evaluation, including comparisons across modeling strategies and clearer reporting of how LLMs are applied.

#### Dataset diversity

One pervasive issue is the lack of validation and generalization of findings across diverse populations. [59] highlighted the need for extensive validation in large-scale studies to confirm the reliability of observed outcomes. This aligns with earlier observations by [29], who pointed to inadequate sample sizes that undermine the statistical power and generalizability of many studies. The representation of diverse populations in studies remains underaddressed, posing a challenge to the equity and applicability of findings. Studies such as those by [50] and [31] emphasized the need to include diverse demographic, cultural, and socioeconomic groups. Without this inclusivity, research risks producing interventions that fail to address the needs of underserved populations, perpetuating health disparities.

Several datasets used in the reviewed studies have known demographic limitations. For example, the MIMIC-III database includes patients from a single Boston hospital and is composed largely of White patients [62]. Similarly, datasets derived from the U.S. Veterans Health Administration predominantly represent older male veterans [63], limiting gender diversity. These characteristics suggest that strong NLP model performance on a specific dataset may not translate to real-world applicability if the underlying data do not adequately represent the broader chronic pain population. Therefore, future research should prioritize external validation of NLP models across institutions and the use of more diverse, multi-institutional datasets, alongside consistent reporting of demographic characteristics to better assess population representativeness and potential biases.

#### Context-specific insights

Mechanistic understanding and context-specific insights also remain underexplored. For instance, [58] stressed the importance of identifying the mechanisms driving intervention efficacy, including the “how" and “why" of their success in specific contexts. [42] further underscored the role of social dynamics in shaping intervention outcomes, which has been largely overlooked. Similarly, [31] highlighted the challenge of differentiating interventions that actively influence outcomes from those merely correlated with behavioral changes. This lack of clarity in mechanisms calls for future studies that delve deeper into contextual and cultural nuances, ensuring that interventions are both targeted and effective for specific subgroups.

#### Standard evaluation metrics

Measurement challenges present another significant barrier. Differences in study design, datasets, and reporting practices make it difficult to compare results across NLP models. While many studies report standard performance measures such as precision, recall, F_1_-score, and accuracy, these metrics are not always used consistently as shown in Table 3.

[45] pointed to difficulties in standardizing metrics, such as those for placebo responses in clinical trials, which complicate cross-study comparisons. [54] emphasized the need to assess the balance of positive versus negative inputs in digital health interventions, a largely neglected area. Furthermore, [53] highlighted the potential of unstructured speech data in chronic pain research, yet its integration into clinical studies remains limited due to lack of methodological tools. Overcoming these challenges requires the development of validated, standardized measurement techniques that enhance consistency across diverse research contexts.

#### Reproducibility

We found that out of all the studies reviewed, only five open-sourced their analysis code-base for other researchers to leverage [31, 32, 38, 41, 44]. This highlights a significant gap in reproducibility and the broader accessibility of methodological advancements. Some studies used datasets containing protected health information (PHI), which limits data sharing. However, it is interesting to note that several PHI-based studies still shared their code repositories, showing that privacy constraints alone do not prevent reproducible research practices.

### Limitations

While the scoping review employed a robust methodology to ensure comprehensive coverage, several limitations should be acknowledged. The reliance on specific databases (PubMed, Web of Science, IEEE Xplore, Scopus, and ACL Anthology) may have excluded relevant studies published in other niche or non-indexed sources, potentially introducing a selection bias. Additionally, the exclusion of certain publication types, such as preprints and gray literature, might have omitted emerging or unconventional perspectives that are not yet peer-reviewed [64]. Furthermore, restricting the search to articles published between 2014 and 2025 may have excluded foundational work or older studies that remain pertinent to the field. The focus broadly on chronic pain likely excluded studies focusing on specific subtypes of chronic pain, since the number of health conditions considered to be chronic pain is fairly large. These limitations should be considered when interpreting the findings of this review.

### Future research directions

#### Cross-institutional validation

NLP has demonstrated its utility as an important tool for characterizing chronic pain-related textual datasets. It will continue to be used for addressing more chronic pain-related research questions and is likely to be used as a default approach along with other approaches (as discussed in Table 3). Overall, the body of evidence highlights the need for methodological rigor, replicable measurement approaches, and interdisciplinary collaboration. Validation of generalizability through large-scale inter-institutional studies and a stronger focus on contextual and cultural sensitivity are critical priorities for future research. Moreover, co-designing NLP tools with provider input can enhance usability and adoption in clinical workflows. Engagement with frontline providers may ensure that outputs from NLP models align with clinical reasoning and documentation practices.

#### Promoting transparency and accessibility

Future researchers should attempt to publicize their code on commonly used version-controlling platforms such as GitHub and Zenodo. Only 14.7% (5/34) of the studies in our review have shared code base [31, 32, 38, 41, 44], and one mentioned the availability upon request [37]. Sharing the codebase may not only allow fellow researchers to reproduce the models and further contribute, but also ensure the reliability of the results presented in the study. Including details like training and test datasets, code to generate models, dependencies used, parameters used, and the computational capacity needed to train models may help other researchers improve upon the existing approaches.

#### Addressing cross-linguistic datasets

Our findings highlight that advanced methods generally achieve higher precision and recall, with some models, like RoBERTa and semantic proximity approaches, achieving F_1_ scores above 0.8. However, certain gaps remain, such as limited attention to cross-linguistic and multimodal datasets and the under-representation of non-English texts. Future research studies should also focus on validating existing (or developing new) models on non-English languages. One potential solution could be training LLMs on diverse cultural and demographic data to create domain-specific corpora that address gaps in socioeconomic, geographic, and linguistic diversity.

#### Temporal analysis integration

Future NLP research should consider incorporating temporal dynamics of pain experiences. Recent findings demonstrate that short-term pain variability exhibits stable patterns that correlate with clinical outcomes [65], suggesting that NLP methods could be enhanced by analyzing how patient-reported pain language evolves over time. This could involve developing models that capture temporal patterns in social media posts, EHR narratives, or patient diaries to provide more comprehensive insights into chronic pain progression and treatment effectiveness.

#### LLMs in future studies

Future research could further increase the use of LLMs by refining multimodal integration (e.g., text and audio), improving literature synthesis with domain-specific training, and ensuring generalizability across chronic pain conditions using larger, diverse datasets. Moreover, dynamic real-time personalization and ethical applications, including bias detection, remain critical areas for advancing LLM use in chronic pain research, bridging current gaps and expanding their clinical impact.

## Conclusions

This review covers the evidence related to NLP systems (including LLMs) for chronic pain over the last decade. The review demonstrates the utility of NLP across various applications such as chronic pain data classification, patient discourse analysis, and treatment outcome prediction. While significant progress has been achieved, several challenges remain, including the need for diverse datasets, standard evaluation metrics, and reproducible research practices. Future efforts should focus on addressing these gaps through interdisciplinary collaboration, methodological rigor, and inclusivity to ensure impactful, equitable, and replicable solutions. These findings not only inform future research directions but also have practical implications for clinicians, who can leverage NLP tools to provide better care for chronic pain patients through a more nuanced understanding of their experiences, risk factors, and treatment responses.

## Supplementary Information

Below is the link to the electronic supplementary material.

Supplementary file 1.

## Data Availability

No datasets were generated or analysed during the current study.

## Appendix A:

Table 4 lists the acronyms and abbreviations used in this paper.

**Table 4:** List of Acronyms

Acronym	Definition	Acronym	Definition	Acronym	Definition
CRIS	Clinical Record Interactive Search	EHR	Electronic Health Records	MIMIC	Medical Information Mart for Intensive Care
GeMAPS	Geneva Minimalistic Acoustic Parameter Set	LDA	Latent Dirichlet Allocation	CBT	Cognitive behavioral therapy
CIH	Complementary and Integrative Health	VHA	Veterans Health Administration	NVDRS	National Violent Death Reporting System
CNN	Convolutional Neural Network	VADER	Valence Aware Dictionary and Sentiment Reasoner	CDW	Corporate Data Warehouse
TF-IDF	Term frequency-inverse document frequency	LBP	Lower Back Pain	GPT	Generative Pre-trained Transformer
ICF	International Classification of Functioning, Disability, and Health	CP	Chronic Pain		

## Appendix B: Search query results

See Table 5.

**Table 5:** Search Strategy Results between 2014 to 2025

S.No.	Database Name	Number of Records
1	PubMed	06
2	Web of Science	41
3	IEEE Xplore	08
4	SCOPUS	67
5	ACL Anthology	33
**Total**		**155**

## References

[1] Cohen SP, Vase L, Hooten WM. Chronic pain: an update on burden, best practices, and new advances. *The Lancet*, 397 (10289): 2082–2097, 2021. ISSN 0140-6736. 10.1016/S0140-6736(21)00393-7. https://www.sciencedirect.com/science/article/pii/S0140673621003937. 34062143

[2] Bauer BA, Tilburt JC, Sood A, Li G, Wang S. Complementary and alternative medicine therapies for chronic pain. Chin J Integr Med. 2016;22(6):403–11. . 10.1007/s11655-016-2258-y 27339090

[3] Stoicea N, Costa A, Periel L, Uribe A, Weaver T, Bergese SD. Current perspectives on the opioid crisis in the us healthcare system: a comprehensive literature review. Medicine (Baltimore). 2019;98(20):e15425. 10.1097/MD.0000000000015425. 31096439 PMC6531094

[4] Stanos S, Brodsky M, Argoff C, et al. Rethinking chronic pain in a primary care setting. Postgrad Med. 2016;128(5):502-515. 10.1080/00325481.2016.1188319. 27166559

[5] Blackwell DL, Lucas JW, Clarke TC . Summary health statistics for U.S. adults: National health interview survey, 2012. Technical Report 260, National Center for Health Statistics, February 2014. https://pubmed.ncbi.nlm.nih.gov/24819891/24819891

[6] Cancer statistics review, 1975-2012 - previous version - seer cancer statistics review. Accessed: 2026-04-12. https://seer.cancer.gov/archive/csr/1975_2012/

[7] Institute of Medicine. *Relieving Pain in America: A Blueprint for Transforming Prevention, Care, Education, and Research*. National Academies Press, June 2011. 10.17226/13172. 22553896

[8] Study shows chronic pain costs united states up to $635 billion. Accessed: 2026-03-15. https://www.socialworktoday.com/news/dn_092112.shtml

[9] De La Rosa JS, Brady BR, Ibrahim MM, Herder KE, Wallace JS, Padilla AR, Vanderah TW. Co-occurrence of chronic pain and anxiety/depression symptoms in U.S. adults: prevalence, functional impacts, and opportunities. *PAIN*, 165 (3): 666, March 2024. ISSN 0304-3959. 10.1097/j.pain.0000000000003056. URL https://journals.lww.com/pain/fulltext/2024/03000/co_occurrence_of_chronic_pain_and.18.aspx. PMC10859853 37733475

[10] Urits I, Schwartz RH, Orhurhu V, Maganty NV, Reilly BT, Patel PM, et al. A comprehensive review of alternative therapies for the management of chronic pain patients: Acupuncture, tai chi, osteopathic manipulative medicine, and chiropractic care. Adv Ther. 2020;38(1):76–89. 10.1007/s12325-020-01554-0. 33184777 PMC7854390

[11] Yang Y, Maher DP, Cohen SP. Emerging concepts on the use of ketamine for chronic pain. Expert Rev Clin Pharmacol. 2020;13(2):135–46. . 10.1080/17512433.2020.1717947 31990596

[12] Brandow AM, Carroll CP, Creary S, et al. American society of hematology 2020 guidelines for sickle cell disease: management of acute and chronic pain. Blood Adv. 2020;4(12):2656-2701. 10.1182/bloodadvances.2020001851. PMC7322963 32559294

[13] Berger AA, Keefe J, Winnick A, et al. Cannabis and cannabidiol (CBD) for the treatment of fibromyalgia. Best Pract Res Clin Anaesthesiol. 2020;34(3):617-631. 10.1016/j.bpa.2020.08.010 33004171

[14] Kinney M, Seider J, Beaty AF, Coughlin K, Dyal M, Clewley D. The impact of therapeutic alliance in physical therapy for chronic musculoskeletal pain: a systematic review of the literature. Phys Therapy Rev. 2018;36(8):886–98. 10.1080/09593985.2018.1516015. 30265840

[15] Dave AD, Ruaño G, Kost J, Wang X. Automated extraction of pain symptoms: A natural language approach using electronic health records. *Pain Phys*, 25 (2): E245–E254, March 2022. https://www.painphysicianjournal.com/current/pdf?article=NzQzMA 35322976

[16] Rajwal S, Pandey AK, Han Z, Sarker A. Unveiling voices: Identification of concerns in a social media breast cancer cohort via natural language processing. In Dina Demner-Fushman, Sophia Ananiadou, Paul Thompson, and Brian Ondov, editors, *Proceedings of the First Workshop on Patient-Oriented Language Processing (CL4Health) @ LREC-COLING 2024*, pp. 264–270, Torino, Italia, May 2024. ELRA and ICCL. https://aclanthology.org/2024.cl4health-1.32/.

[17] Guo Y, Rajwal S, Lakamana S, Chiang CC, Menell PC, Shahid AH, Chen Y-C, Chhabra N, Chao W-J, Chao C-J, et al. Generalizable natural language processing framework for migraine reporting from social media. *AMIA Summits on Translational Science Proceedings*, 2023: 261, 2023. https://pmc.ncbi.nlm.nih.gov/articles/PMC10283091/. PMC10283091 37350878

[18] Sarker A, Lakamana S, Guo Y, Ge Y, Leslie A, Okunromade O, Gonzalez-Polledo E, Perrone J, McKenzie-Brown AM. #ChronicPain: Automated Building of a Chronic Pain Cohort from Twitter Using Machine Learning. *Health Data Science*, 3: 0078, January 2023. ISSN 2765-8783. 10.34133/hds.0078. https://spj.science.org/doi/10.34133/hds.0078. PMC10852024 38333075

[19] Bacco L, Russo F, Ambrosio L, D’Antoni F, Vollero L, Vadalà G, Dell’Orletta F, Merone M, Papalia R, Denaro V. Natural language processing in low back pain and spine diseases: A systematic review. *Frontiers in Surgery*, 9, July 2022. ISSN 2296-875X. 10.3389/fsurg.2022.957085. https://www.frontiersin.org/journals/surgery/articles/10.3389/fsurg.2022.957085. Publisher: Frontiers. PMC9329654 35910476

[20] Norel R, Gewandter J, Zhang Z, Tahsin A, Abdallah CG, Markman J, Duan Z, Cecchi G, Geha P. Turning Patients’ Open-Ended Narratives of Chronic Pain Into Quantitative Measures: Natural Language Processing Study. JMIR human factors. 2025 Nov 25;12(1):e80269. 10.2196/80269. PMC12690277 41290220

[21] Mikolov T, Chen K, Corrado G, Dean J. Efficient estimation of word representations in vector space, 2013. arXiv: 1301.3781v3.

[22] Vaswani A, Shazeer N, Parmar N, Uszkoreit J, Jones L, Gomez AN, Kaiser L, Polosukhin I. Attention Is All You Need. 2023. 10.48550/arXiv.1706.03762. http://arxiv.org/abs/1706.03762

[23] Devlin J, Chang M-W, Lee K, Toutanova K. BERT: Pre-training of Deep Bidirectional Transformers for Language Understanding, 2019. arXiv:1810.04805 [cs].

[24] Radford A, Narasimhan K, Salimans T, Sutskever I. Improving Language Understanding by Generative Pre-Training. 2018. https://www.semanticscholar.org/paper/Improving-Language-Understanding-by-Generative-Radford-Narasimhan/cd18800a0fe0b668a1cc19f2ec95b5003d0a5035.

[25] Tricco AC, Lillie E, Zarin W, O’Brien KK, Colquhoun H, Levac D, et al. Prisma extension for scoping reviews (prisma-scr): checklist and explanation. Ann Intern Med. 2018;169(7):467–73. 10.7326/m18-0850 30178033

[26] JBI Manual for Evidence Synthesis - JBI Global Wiki. https://jbi-global-wiki.refined.site/space/MANUAL.

[27] Sterne JAC, Hernán MA, Reeves BC, Savović J, Berkman ND,Viswanathan M, Henry D, Altman DG, Ansari MT, Boutron I, Carpenter JR, Chan A-W, Churchill R, Deeks JJ, Hróbjartsson A, Kirkham J, Jüni P, Loke YK, Pigott TD, Ramsay CR, Regidor D, Rothstein HR, Sandhu L, Santaguida PL, Schünemann HJ, Shea B, Shrier I, Tugwell P, Turner L, Valentine JC, Waddington H, Waters E, Wells GA, Whiting PF, Higgins JPT. ROBINS-I: a tool for assessing risk of bias in non-randomised studies of interventions. *BMJ*, 355: i4919, October 2016. ISSN 1756-1833. 10.1136/bmj.i4919. https://www.bmj.com/content/355/bmj.i4919. Publisher: British Medical Journal Publishing Group Section: Research Methods & Reporting. PMC5062054 27733354

[28] Hylan TR, Von Korff M, Saunders K, Masters E, Palmer RE, Carrell D, et al. Automated prediction of risk for problem opioid use in a primary care setting. J Pain. 2015;16(4):380–7. 10.1016/j.jpain.2015.01.011. Publisher: Churchill Livingstone Inc 25640294

[29] Yang Z, Dehmer M, Yli-Harja O, Emmert-Streib F. Combining deep learning with token selection for patient phenotyping from electronic health records. *Sci. Rep.*, 10 (1), 2020b. 10.1038/s41598-020-58178-1. Publisher: Nature Research. PMC6989657 31996705

[30] Dobscha SK, Luther SL, Kerns RD, Finch DK, Goulet JL, Brandt CA, et al. Mental Health Diagnoses are Not Associated With Indicators of Lower Quality Pain Care in Electronic Health Records of a National Sample of Veterans Treated in Veterans Health Administration Primary Care Settings. J Pain. 2023;24(2):273–81. 10.1016/j.jpain.2022.08.009. Publisher: Elsevier B.V PMC9898089 36167230

[31] Chaturvedi J, Stewart R, Ashworth M, Roberts A. Distributions of recorded pain in mental health records: A natural language processing based study. *BMJ OPEN*, 14(4), 2024. 10.1136/bmjopen-2023-079923. PMC11033644 38642997

[32] Zhao X, Liu S, Yang S-Y, Miao C. MedRAG: Enhancing Retrieval-augmented Generation with Knowledge Graph-Elicited Reasoning for Healthcare Copilot. pages 4442–4457, 2025. 10.1145/3696410.3714782.

[33] Gordon KS, Buta E, Pratt-Chapman ML, Brandt CA, Gueorguieva R, Warren AR, et al. Relationship Between Pain and LGBT Status Among Veterans in Care in a Retrospective Cross-Sectional Cohort. J Pain Res. 2023;16:4037–47. Publisher: Dove Medical Press Ltd 10.2147/JPR.S432967 PMC10695019 38054108

[34] Ashar YK, Lumley MA, Perlis RH, Liston C, Gunning FM, Wager TD. Reattribution to mind-brain processes and recovery from chronic back pain: a secondary analysis of a randomized clinical trial. JAMA Netw Open. 2023;6(9):e2333846. 10.1001/jamanetworkopen.2023.33846. 37768666 PMC10539987

[35] Cintron DW, Ong AD, Reid MC. What makes life go well? A network topic modeling analysis of well-being practices in adults with chronic pain. Pain Medicine (United States). 2025;26(4):189–98. 10.1093/pm/pnae131 PMC11967178 39724365

[36] Haller IV, Renier CM, Juusola M, Hitz P, Steffen W, Asmus MJ, et al. Enhancing risk assessment in patients receiving chronic opioid analgesic therapy using natural language processing. Pain Med. 2017;18(10):1952–60. 10.1093/pm/pnw283. Publisher: Oxford University Press 28034982

[37] Comito C, Forestiero A, Macrì D, Metlichin E, Giusti GD, Ramacciati N. Comparative analysis of AI algorithms on real medical data for chronic pain detection. *Int J Med Inform*, 203: 106002 10.1016/j.ijmedinf.2025.106002. https://www.sciencedirect.com/science/article/pii/S1386505625002199 40505249

[38] Elyazori HRA, Abdulrazzaq R, Shawi HA, Amouzou I, King P, Manns S, Popal M, Patel Z, Destefano S, Shah J, Gerber N, Sikdar S, Lee S, Acuna S, Lybarger K. Capturing patients’ lived experiences with chronic pain through motivational interviewing and information extraction. In Sophia Ananiadou, Dina Demner-Fushman, Deepak Gupta, and Paul Thompson, editors, *Proceedings of the Second Workshop on Patient-Oriented Language Processing (CL4Health)*, pages 321–330, Albuquerque, New Mexico, May 2025. Association for Computational Linguistics. https://aclanthology.org/2025.cl4health-1.28/

[39] Amidei J, Nieto R, Kaltenbrunner A, de Sá JGF, Serrat M, Albajes K. Exploring the Capacity of Large Language Models to Assess the Chronic Pain Experience: Algorithm Development and Validation. *Journal of Medical Internet Research*, 27, 2025. 10.2196/65903 PMC11997525 40163858

[40] Goudman L, De Smedt A, Moens M. Social Media and Chronic Pain: What Do Patients Discuss? *J. Pers. Med.*, 12 (5), 2022. 10.3390/jpm12050797 PMC9146886 35629218

[41] Nunes DAP, Ferreira-Gomes J, Neto F, Martins de Matos D. Modeling Chronic Pain Experiences from Online Reports Using the Reddit Reports of Chronic Pain Dataset. *Information*, 14 (4), 2023a. Publisher: MDPI. 10.3390/info14040237

[42] Necaise A, Amon MJ. Peer Support for Chronic Pain in Online Health Communities: Quantitative Study on the Dynamics of Social Interactions in a Chronic Pain Forum. *J. Med. Internet Res.*, 26, 2024. Publisher: JMIR Publications Inc. 10.2196/45858 PMC11413547 39235845

[43] Bozkurt S, Walker A, Park T, Le N, Sarker A, Irani A, et al. The Digital Narrative: Chronic Pain Self-Management Insights from Reddit in Systemic Lupus Erythematosus. In Envisioning the Future of Health Informatics and Digital Health. 2025;323:483–5. 10.3233/SHTI250137 40200534

[44] Chaturvedi J, Mascio A, Velupillai SU, Roberts A. Development of a Lexicon for Pain. *Front. Digit. Health*, 3, 2021. Publisher: Frontiers Media SA. 10.3389/fdgth.2021.778305 PMC8710455 34966903

[45] Berger SE, Branco P, Vachon-Presseau E, Abdullah TB, Cecchi G, Vania Apkarian A. Quantitative language features identify placebo responders in chronic back pain. *Pain*, 162 (6): 1692–1704, 2021. 10.1097/j.pain.0000000000002175. Publisher: Lippincott Williams and Wilkins. 33433145

[46] Nunes DAP, Ferreira-Gomes J, Oliveira D, Vaz C, Pimenta S, Neto F, de Matos DM. Chronic Pain Patient Narratives Allow for the Estimation of Current Pain Intensity. *2023 IEEE 36th International Symposium on Computer-Based Medical Systems (CBMS)*, 12 (NA): 716–719, 2023b. 10.1109/cbms58004.2023.00306.

[47] Branco P, Berger S, Abdullah T, Vachon-Presseau E, Cecchi G, Vania Apkarian A. Predicting placebo analgesia in patients with chronic pain using natural language processing: a preliminary validation study. *PAIN*, 164 (5): 1078, May 2023. ISSN 0304-3959. 10.1097/j.pain.0000000000002808 PMC10106359 36524810

[48] Aggarwal A, Rai S, Giorgi S, et al. A Cross-Modal Study of Pain Across Communities in the United States. Association for Computing Machinery; 2023:1050-1058. 10.1145/3543873.3587642.

[49] Davidson JE, Ye G, Parra MC, Choflet A, Lee K, Barnes A, et al. Job-Related Problems Prior to Nurse Suicide, 2003–2017: A Mixed Methods Analysis Using Natural Language Processing and Thematic Analysis. J Nurs Regul. 2021;12(1):28–39. 10.1016/S2155-8256(21)00017-X. Publisher: Elsevier Inc

[50] Taylor SL, Herman PM, Marshall NJ, Zeng Q, Yuan A, Chu K, Shao Y, Morioka C, Lorenz KA. Use of Complementary and Integrated Health: A Retrospective Analysis of U.S. Veterans with Chronic Musculoskeletal Pain Nationally. *J. Altern. Complement. Med.*, 25 (1): 32–39, 2019. 10.1089/acm.2018.0276. Publisher: Mary Ann Liebert Inc. 30312109

[51] Goldstein EV, Mooney SJ, Takagi-Stewart J, Agnew BF, Morgan ER, Haviland MJ, et al. Characterizing Female Firearm Suicide Circumstances: A Natural Language Processing and Machine Learning Approach. Am J Prev Med. 2023;65(2):278–85. 10.1016/j.amepre.2023.01.030. Publisher: Elsevier Inc 36931986

[52] Schirle L, Jeffery A, Yaqoob A, Sanchez-Roige S, Samuels DC. Two data-driven approaches to identifying the spectrum of problematic opioid use: A pilot study within a chronic pain cohort. *Int. J. Med. Informatics*, 156, 2021. 10.1016/j.ijmedinf.2021.104621. Publisher: Elsevier Ireland Ltd. PMC8609775 34673309

[53] Agurto C, Merler M, Reinen J, Parida P, Cecchi G, Rogers JL. Exploring chronic pain experiences: leveraging text and audio analysis to infer well-being metrics. pp 196–201, 2024. 10.1109/ICDH62654.2024.00041.

[54] Reinen JM, Agurto C, Cecchi G, Rogers JL. Remotely-captured, free-text responses track with patient health states in chronic pain. pages 169–171, 2024. 10.1109/ICDH62654.2024.00037.

[55] Lin D, Koenig C, Kaplan S, Bittner M, Paraiso M, Buhn N, Stokes E, Ho I, Lotz J, Davidson J. Investigating the Impact of Context on Zero-Shot Entity Resolution: Applications in Chronic Lower Back Pain. pp 390–396, 2025. 10.1109/ICICT64582.2025.00067.

[56] Chaturvedi J, Chance N, Mirza L, et al. Development of a Corpus Annotated With Mentions of Pain in Mental Health Records: Natural Language Processing Approach. JMIR Form Res. 2023;7:e45849. 10.2196/45849. PMC10337440 37358897

[57] Lotz JC, Ropella G, Anderson P, et al. An exploration of knowledge-organizing technologies to advance transdisciplinary back pain research. JOR Spine. 2023;6(4):e1300. 10.1002/jsp2.1300. PMC10751978 38156063

[58] Chen AT, Swaminathan A, Kearns WR, Alberts NM, Law EF, Palermo TM. Understanding user experience: Exploring participants messages with a web-based behavioral health intervention for adolescents with chronic pain. J Med Internet Res. 2019;21(4):e11756. 10.2196/11756 PMC6487347 30985288

[59] Venerito V, Iannone F. Large language model-driven sentiment analysis for facilitating fibromyalgia diagnosis. RMD Open. 2024;10(2):e004367. 10.1136/rmdopen-2024-004367 PMC11227845 38942593

[60] Himmelstein G, Bates D, Zhou L. Examination of Stigmatizing Language in the Electronic Health Record. JAMA Netw Open. 2022;5(1):e2144967. Publisher: American Medical Association. 10.1001/jamanetworkopen.2021.44967 PMC8796019 35084481

[61] Liu Y, Ott M, Goyal N, et al. RoBERTa: A Robustly Optimized BERT Pretraining Approach. Preprint. 2019. arXiv:1907.11692.

[62] Alistair EW Johnson, Tom J Pollard, Lu Shen, Li-wei H Lehman, Mengling Feng, Mohammad Ghassemi, Benjamin Moody, Peter Szolovits, Leo Anthony Celi, and Roger G Mark. Mimic-iii, a freely accessible critical care database. *Scientific data*, 3 (1): 1–9, 2016. 10.1038/sdata.2016.35. PMC4878278 27219127

[63] *National Healthcare Quality and Disparities Report: Chartbook on Healthcare for Veterans*. Agency for Healthcare Research and Quality (US), Rockville, MD, November 2020. URL https://www.ncbi.nlm.nih.gov/books/NBK578553/. Available from: National Center for Biotechnology Information (NCBI) Bookshelf. 35263061

[64] Adams J, Hillier-Brown FC, Moore HJ, et al. Searching and synthesising 'grey literature' and 'grey information' in public health: critical reflections on three case studies. Syst Rev. 2016;5(1):164. 10.1186/s13643-016-0337-y. PMC5041336 27686611

[65] Zheng X, Rajwal S, Ashworth C, et al. Short-term variability of chronic musculoskeletal pain. Front Pain Res. 2025;6:1626589. 10.3389/fpain.2025.1626589 PMC12460470 41019708

